# Simulating social-ecological systems: the Island Digital Ecosystem Avatars (IDEA) consortium

**DOI:** 10.1186/s13742-016-0118-5

**Published:** 2016-03-17

**Authors:** Neil Davies, Dawn Field, David Gavaghan, Sally J. Holbrook, Serge Planes, Matthias Troyer, Michael Bonsall, Joachim Claudet, George Roderick, Russell J. Schmitt, Linda Amaral Zettler, Véronique Berteaux, Hervé C. Bossin, Charlotte Cabasse, Antoine Collin, John Deck, Tony Dell, Jennifer Dunne, Ruth Gates, Mike Harfoot, James L. Hench, Marania Hopuare, Patrick Kirch, Georgios Kotoulas, Alex Kosenkov, Alex Kusenko, James J. Leichter, Hunter Lenihan, Antonios Magoulas, Neo Martinez, Chris Meyer, Benoit Stoll, Billie Swalla, Daniel M. Tartakovsky, Hinano Teavai Murphy, Slava Turyshev, Fernanda Valdvinos, Rich Williams, Spencer Wood, IDEA Consortium

**Affiliations:** Gump South Pacific Research Station, University of California Berkeley, Moorea, BP 244 98728 French Polynesia; Biodiversity Institute, Department of Zoology, University of Oxford, The Tinbergen Building, South Parks Road, Oxford, OX1 3PS UK; Berkeley Institute for Data Science,190 Doe Library, University of California, Berkeley, CA 94720 USA; Computational Biology Group, Department of Computer Science, University of Oxford, Wolfson Building, Oxford, UK; Department of Ecology, Evolution and Marine Biology and the Marine Science Institute, University of California Santa Barbara, Santa Barbara, CA 93106 USA; Laboratoire d’Excellence CORAIL, USR 3278 CNRS—EPHE—UPVD, Centre de Recherche Insulaire et Observatoire de l’Environnement (CRIOBE), Papetoai, Moorea BP 1013 - 98 729 French Polynesia; Institute for Theoretical Physics and Platform for Advanced Scientific Computation, ETH Zurich, Zurich, 8093 Switzerland; Department of Environmental Science, Policy, & Management, 130 Mulford Hall #3114, University of California, Berkeley, CA 94720 USA; The Josephine Bay Paul Center for Comparative Molecular Biology and Evolution, Marine Biological Laboratory, Woods Hole, MA 02543 USA; Unit of Medical Entomology, Institut Louis Malardé, Tahiti, BP 30, 98713 French Polynesia; Ecole Pratique des Hautes Etudes, Laboratory of Coastal Geomorphology and Environment, Dinard, France; Berkeley Natural History Museums, 3101 Valley Life Sciences Building, Berkeley, CA 94720 USA; National Great Rivers Research and Education Center (NGRREC), One Confluence Way, East Alton, IL 62024 USA; Santa Fe Institute, 1399 Hyde Park Road, Santa Fe, NM 87501 USA; Hawaii Institute of Marine Biology, School of Ocean & Earth Science & Technology, University of Hawaii at Manoa, PO Box 1346, Kaneohe, HI 96744 USA; United Nations Environment Programme World Conservation Monitoring Centre, 219 Huntingdon Road, Cambridge, CB3 0DL UK; Marine Laboratory, Nicholas School of the Environment, Duke University, 135 Marine Lab Road, Beaufort, NC 28516 USA; Laboratoire GePaSud, Université de la Polynésie Française, Tahiti, BP6570, 98702 Faa’a French Polynesia; Department of Anthropology, University of California, 232 Kroeber Hall, Berkeley, CA 94720 USA; Institute of Marine Biology, Biotechnology and Aquaculture Hellenic Centre for Marine Research Gournes Pediados, PO Box 2214, Heraklion, Crete GR 710 03 Greece; Department of Physics and Astronomy, University of California, 475 Portola Plaza, Los Angeles, CA 90095 USA; Scripps Institution of Oceanography, University of California San Diego, 9500 Gilman Drive, La Jolla, CA 92093 USA; Bren School of Environmental Science and Management, 3428 Bren Hall, University of California, Santa Barbara, CA 93106 USA; Department of Ecology and Evolutionary Biology, University of Arizona, Tucson, AZ 85721 USA; Pacific Ecoinformatics and Computational Ecology Lab, Berkeley, CA 94703 USA; Department of Invertebrate Zoology, National Museum of Natural History, Smithsonian Institution, PO Box 37012, MRC-163, Washington, DC 20013 USA; Friday Harbor Laboratories, University of Washington, 620 University Road, Friday Harbor, WA 98250 USA; Department of Mechanical and Aerospace Engineering, University of California San Diego, 9500 Gilman Drive, Mail Code 0411, La Jolla, CA 92093 USA; Atitia Center, Gump Station, University of California Berkeley, Moorea, BP 244 98728 French Polynesia; NASA Jet Propulsion Laboratory, California Institute of Technology, 4800 Oak Grove Drive, Pasadena, CA 91109 USA; Department of Physics and Astronomy and Department of Earth and Planetary Sciences University of California, Los Angeles, CA 90095 USA; Vibrant Data Inc., 943 Clay Street, San Francisco, Calfornia 94108 USA; School for Environmental and Forest Sciences, University of Washington, Box 352100, Seattle, Washington 98195 USA; IDEA Consortium, ETH Zurich, Zurich, Switzerland; http://MooreaIDEA.org/participants

**Keywords:** Computational ecology, Biodiversity, Genomics, Biocode, Earth observations, Social-ecological system, Ecosystem dynamics, Climate change scenarios, Predictive modeling

## Abstract

Systems biology promises to revolutionize medicine, yet human wellbeing is also inherently linked to healthy societies and environments (sustainability). The IDEA Consortium is a systems ecology open science initiative to conduct the basic scientific research needed to build use-oriented simulations (avatars) of entire social-ecological systems. Islands are the most scientifically tractable places for these studies and we begin with one of the best known: Moorea, French Polynesia. The Moorea IDEA will be a sustainability simulator modeling links and feedbacks between climate, environment, biodiversity, and human activities across a coupled marine–terrestrial landscape. As a model system, the resulting knowledge and tools will improve our ability to predict human and natural change on Moorea and elsewhere at scales relevant to management/conservation actions.

## Background

High-throughput data collection techniques and large-scale computing are transforming our understanding of ecosystems, making convergent scientific frameworks a research priority [1]. As human activities increasingly impact ecosystem processes, we need new approaches that focus on how whole communities of organisms interact with people and the physical environment at the scale of landscapes or catchments [2]. This requires an e-infrastructure for data intensive science that enables the integration of computational physics, chemistry, biology, ecology, economics and other social sciences. Such an advance would allow researchers to (1) characterize the multidisciplinary functional attributes of social-ecological systems; (2) quantify the relationships between those functional attributes under historic and current conditions; and (3) model the trajectories of goods and services under a range of policy-driven scenarios and future environmental conditions. The resulting knowledge would improve our ability to predict human and natural change at scales relevant to management/conservation actions.

Unlike some aspects of climate change, processes related to biodiversity and ecosystem services are “typically place-based and many of the effects are seen at sub-global scales” [3]. Inspired by successes in modeling complex systems at other scales of organization, notably the cell [4], the Island Digital Ecosystem Avatars (IDEA) Consortium aims to build computer simulations (‘avatars’) to the scale of whole social-ecological systems. With a common boundary constraining their physical, ecological, and social networks, islands have long been recognized as model systems for ecology and evolution [5]. Their geography sets clear limits on the species to inventory, space holders (ground cover) to measure, organisms to count, physical–chemical contexts to characterize, and natural–human interactions to consider. The knowledge and cyberinfrastructure developed for island avatars, and complementary efforts targeting major cities like Singapore [6] and New York [7], will eventually scale to countries and regions, including their associated coastal waters. Island avatars are built from the genome up, while at the same time downscaling regional models to establish boundary conditions. They address many of the challenges faced by macrosystems ecology [8], and general ecosystem models [9]. Indeed, an Earth Avatar would converge on the Global Earth Observation System of Systems (GEOSS) [10] and Future Earth [11]. The IDEA approach, however, avoids overwhelming complexity, instead concentrating effort on the simplest social–ecological systems that include most of the data types covered by GEOSS, and many of the processes found globally.

### Moorea IDEA

The small island of Moorea (134 km^2^) in French Polynesia is well placed for a proof-of-concept study. About 15 km northwest of Tahiti with a population of ~17,000, Moorea is perhaps the best studied island in the world [12] thanks to several decades of activity at its two research stations (CNRS-EPHE CRIOBE [13], and the University of California (UC) Berkeley Gump Station [14]), which respectively house France’s Center of Excellence for Coral Reef Research (LabEx CORAIL [15]), and the US National Science Foundation’s only coral reef Long-Term Ecological Research (MCR LTER) site [16], which is administered by UC Santa Barbara. Additionally, the Moorea Biocode Project [17] has characterized every species (>1 mm) on the island, including genetic sequences, museum specimens, and digital photographs [18]. While there is still much more to learn, especially concerning the vastly diverse microbes [19] and human systems, the existing physical and biotic databases provide a powerful foundation for whole system ecological modeling. Combined with a wealth of data on the resilience of Moorea’s ecosystems, including the response of its coral reefs to large-scale perturbations [20] and the evolution of Polynesian society [21], Moorea has many of the characteristics needed to advance systems ecology and sustainability science [22].

**Fig. 1:**
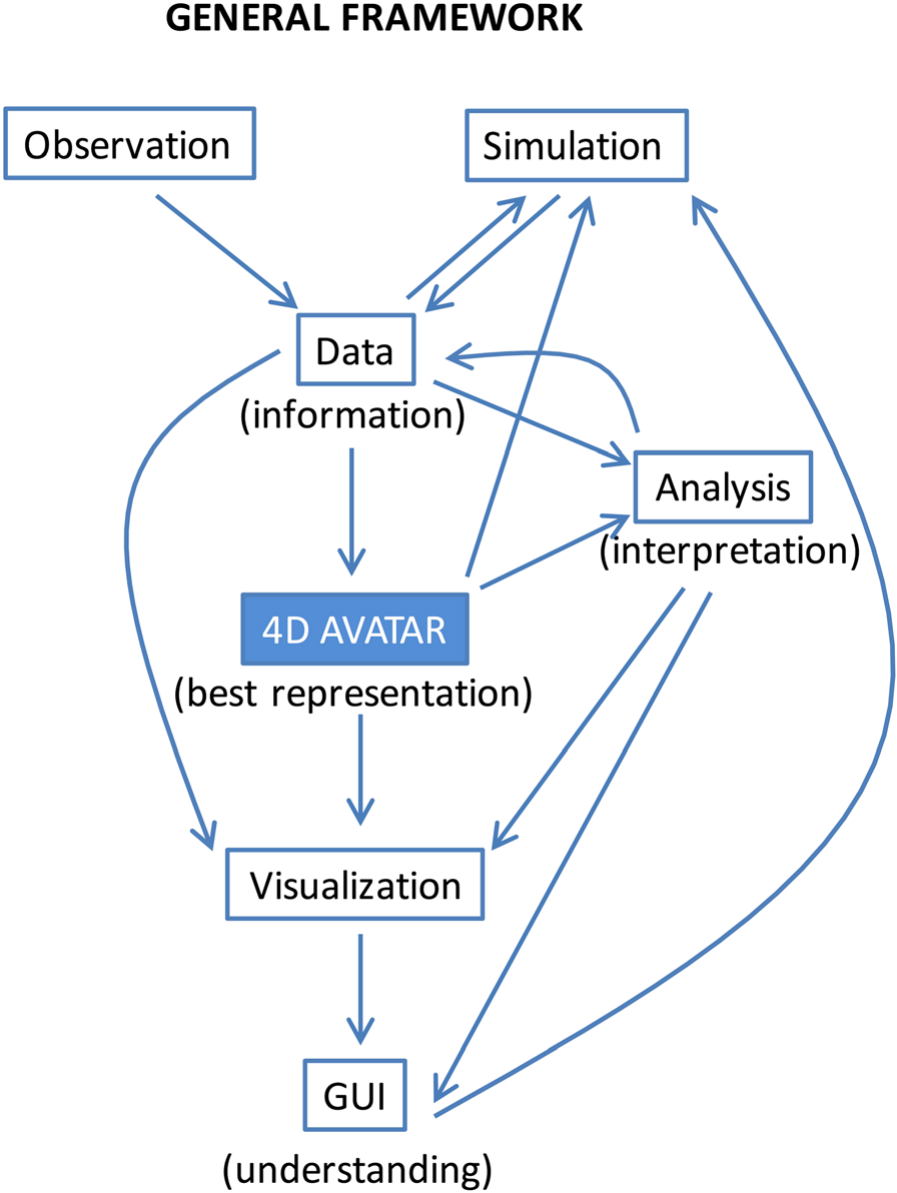
The data-driven avatar communicates knowledge through a Graphical User Interface (GUI) customized for different stakeholders. Their improved understanding in turn generates new hypotheses to test and new simulations to run, thus driving further data collection

The Moorea IDEA aims to understand how biodiversity, ecosystem services, and society will co-evolve over the next several decades depending upon what actions are taken. Specifically, we ask: (1) what is the physical, biological, and social state of the island system today? (2) How did it get to this point? (3) What is its future under alternative scenarios of environmental change and human activity, including conservation efforts? These questions are addressed through a place-based data science infrastructure and computational platform (Fig. 1). The Moorea Avatar is the best digital representation of the island; a three-dimensional visualization of Moorea that looks similar to the one on Google Earth, but which includes the dimension of time and enables researchers to zoom into a location, access data, and run simulations. Today’s island represents the key baseline because the majority of the modeling data needed are not available for historic time periods. The Avatar computational platform allows other versions of Moorea to be generated and visualized *in silico* for a range of purposes. This involves the integration of physical, biological, and social data [12–21], and drawing on best-available scientific knowledge to show what the island looked like in the past (using historic baselines to explore particular issues), and to predict how it might look in the future. Unlike video games, our projections are constrained by reality and are intended not only for research and education, but also to support scenario-based planning; helping local communities adapt to environmental change and maximize ecological resilience. Our goal is to emulate P4 Medicine, extending to social–ecological–physical systems the Predictive, Preventive, Personalized, and Participatory approach that promises to revolutionize the biomedical field [23].

Working with the local population to co-develop island avatars as “boundary objects” [24] is critical to ensuring that the simulations are useful, credible and legitimate. Non-scientist stakeholders take joint ownership of their avatar by prioritizing the policies to simulate (e.g., conservation plans), and by contributing traditional/local knowledge and data. The participatory approach includes citizen science, taking advantage of newly affordable technologies and helping reconnect people with natural processes through observation and experiential learning.

### Organization

The Moorea IDEA was initiated by researchers associated with the UC Berkeley Gump Research Station and CRIOBE, through a workshop at the Pauli Center for Theoretical Studies, ETH Zurich, in November 2013. The Consortium now includes more than 80 scientists from around the world, representing the physical, biological, social, computer and information sciences. Institutional nodes (more than 20) form its governing body, which is currently led by an Executive Committee representing the founding institutions. The Consortium addresses convergent research questions across areas of societal interest (energy, water, nutrients, biodiversity, food and nutrition, and health) through five overlapping and interlinked working groups (Table 1). The Moorea IDEA has gained support from the Municipality of Moorea and the territorial government of French Polynesia. In the future, other islands could join to form a network that shares the generally applicable tools and approaches generated by the IDEA Consortium. For example, the nearby atoll Tetiaroa constitutes a worthwhile comparison to Moorea. Further afield, and reflecting the ambition to scale to larger places, scientists and government officials from Crete have expressed a strong interest in the project.

**Table 1:** Research priorities of the IDEA Consortium

Working group	Task
1. Data science	Integrating diverse data sources, coupling models, and visualizing information
2. Physical modeling	Oceanic and atmospheric forcing, and physical-chemical properties and fluxes
3. Genes to ecosystems	Biodiversity dynamics, evolutionary processes, and ecological interactions
4. Social-ecological systems	Coupling past, present, and future ecosystems to human activities
5. Simulations, synthesis, and service	Use-oriented avatar as a platform for data exploration, scenario-based planning, and education

## Conclusions

The new Intergovernmental Platform on Biodiversity and Ecosystem Services (IPBES) has prioritized an assessment on the “Modeling of Biodiversity and Ecosystem Services” [25]. Addressing this grand challenge will require computational models of place that are able to simulate alternative scenarios and visualize likely outcomes for scientists, policymakers, and the public [26]. Big data, computational ecology, and sophisticated simulation platforms cannot solve all of the world’s problems, but harnessing scalable technology addresses the lack of capacity in local knowledge management systems, and can help illuminate pathways to sustainability. In an era in which society is seeking to transition to clean energy and sustainable economic growth, knowledge of the pathways to these futures is needed, along with showcases demonstrating that such change is possible. At least initially, examples are more likely to come from islands and cities than large regions and countries. Islands are disproportionately affected by global change and epitomize the coastal zones where most of humanity lives. They serve as models for continental regions and, ultimately, for our common island home: planet Earth.

## Abbreviations

CRIOBE: Centre de Recherches Insulaires et Observatoire de l’Environnement (Center for Island Research and Environmental Observatory)
GEO: Group on earth observations
GEOSS: Global Earth Observation System of Systems
IDEA: Island Digital Ecosystem Avatars
IPBES: International panel on biodiversity and ecosystem services
LTER: Long Term Ecological Research
